# Synergistic enhancing of micellization and thermodynamic properties of some Gemini cationic surfactants related to benzo[d]thiazol-3-ium bromide

**DOI:** 10.1186/s13065-024-01334-9

**Published:** 2024-12-11

**Authors:** Farid I. El-Dossoki, Mohamed A. Migahed, Mahmoud M. Gouda, Samir A. Abd El-Maksoud

**Affiliations:** 1 Chemistry Department, Faculty of Science, Port-Said University, Port-Said, Egypt; 2 Department of Petroleum Applications, Egyptian Petroleum Research Institute (EPRI), Cairo, Egypt

**Keywords:** Gemini cationic surfactants, Micellization, Conductivity, Polarizability

## Abstract

Herrin, three Gemini cationic surfactants related to benzo[d]thiazol-3-ium bromide with variable hydrocarbon chain lengths (TBC n = 6, 12, and 18) were synthesized successfully and confirmed by using IR and ^1^HNMR spectroscopies. Critical micelle concentration and different thermodynamic properties of all surfactants under study were measured using conductivity, density, molal volume, and refractive index techniques. The Critical micelle concentration of TBC 6, TBC 12, and TBC 18 surfactants measured from the different techniques shows an acceptable agreement. The molecular weight of the investigated surfactants was decreased with the order: TBC 18 > TBC 12 > TBC 6. An increase in the magnitudes of the association constant, Gibbs free energy of micellization, molar refraction, polarizability, and binding constant proved the effect of hydrocarbon chain length on increasing surfactant’s micellization as follows: TBC 18 < TBC 12 < TBC 6. The enhancement in surfactant properties was also indicated under the effect of different concentrations of inorganic salts (NaI, NaBr, NaCl, MnCl_2_, CuCl_2,_ and CoCl_2_). This effect was measured using conductivity and refractive index measurements. Different salts were indicated to adsorb on head groups of micelles, leading to an increase in the degree of ionization of the surfactant solution and improved aggregation of the surfactant at lower concentrations. The increase in the negative value of Gibbs free energy of association in the presence of salts proved an increase in the stability of micelles formed in a 15% DMSO-water solvent at 298.15 K.

**Supplementary Information:**

The online version contains supplementary material available at 10.1186/s13065-024-01334-9.

## Introduction

Gemini cationic surfactants have gained much attention in several fields, including detergents, coatings, biocides, pharmacy, food processing, enhanced oil recovery, and environmental protection [1–4]. By doubling the hydrophilic head and hydrophobic tail, Gemini cationic surfactants have higher surface activity and a lower critical micelle concentration [5]. The hydrophilic heads were separated with spacers with different compositions [6]. Aggregation is induced by attraction between the hydrophobic tails, while an interfacial area is ensured by repulsion between the hydrophilic head groups [7]. By altering the hydrophobic tail's length and the head groups and adjusting this force balance between opposing forces, the micellization of surfactant can be tuned [8, 9].

The critical micelle concentration (CMC) is what determines how effective surfactants are. When any of the physicochemical properties are plotted versus surfactant concentration, an inflection point can be found that represents the minimal range of concentration at which the surfactant monomer begins to self-associate [10–12]. Several direct techniques used to measure critical micelle concentrations of surfactants include surface tension, conductivity, osmotic pressure, refractive index, and viscosity [13–18]. Also, CMC may be measured indirectly by volumetric and spectrometric methods [16, 19].

Adding inorganic salts to surfactant solutions increases their importance in several emulsifications in the industry. Salting-out frequently occurs when surfactant and salt are combined in a solution. The preferential migration of water molecules from the coordination shells of surfactant molecules to those of salts, which immobilizes and quenches their activity as solvents, is the cause of salting-out, according to hydration theory [20–24]. The inorganic salts affect the morphology of surfactants leading to increased hydrophobicity [9].

Thermodynamic parameters were indicated, such as the degree of ionization, binding constant, Gibbs free energy of micellization and association, molar refraction, atomic polarizability, Van der Waals volume, and electrostriction volume [25–27]. Properties of all surfactants were indicated to change under the effect of different inorganic salts at different concentrations [20, 23, 28]. Salts are indicated to improve micellization of surfactants in solution at a lower concentration [28–31]. Improvements in the properties of surfactants were computed related to solvation parameters, including the binding constant of counter ions and Gibbs free energy of micellization and association from conductivity technique [32, 33]. Changes in refractive indices proved the enhancement in the micellization process in the presence of salts.

In this study, the synthesis of three new cationic Gemini surfactants from the reaction between terephthalaldehyde and benzo[d]thiazole-2amine, which followed a reaction with various alkyl halides, was discussed. The structure of all surfactants was confirmed using ^1^H-NMR and IR spectroscopy. The critical micelle concentration of all surfactants was measured using conductivity, refractive index, density, and molar volume techniques. Various thermodynamic parameters related to the mentioned techniques were discussed. For all surfactants for which parameter changes were explained, the effect of two different concentrations of six inorganic salts was reported. All measurements were reported in 15% dimethyl sulfoxide (DMSO)-water solvent.

## Experimental

### Materials

All materials used in this study were selected with a high purity value indicated in a mass percent as follows: terephthaladehyde (99%), benzo[d]thiazol-2-amine (99%), bromohexane (98%), bromododecane (97%), bromooctadecane (97%), acetone (99.9%), ethanol (99.9%), sodium chloride (98%), sodium bromide (98%), sodium iodide (98%), cobalt chloride (98%), manganese chloride (98%) and copper chloride (98%). All chemicals were purchased from the international Sigma Aldrich company, United States without needing any purification. Distilled water used in this study had a conductivity of less than 2 µS cm^−1^.

### Synthesis of TBC Gemini cationic surfactant

**Scheme 1:**
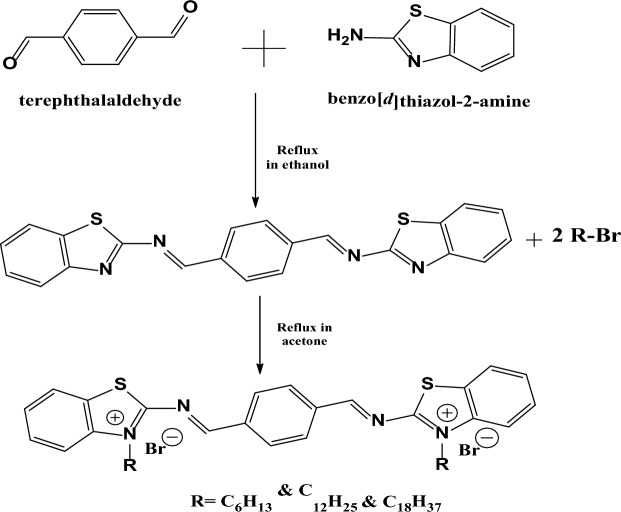
Synthetic Route of TBC Gemini Cationic Surfactants

Terphtalaldehye (0.1 mol) was refluxed with 0.1 mol of benzo[d]thiazol-2amine for 12 h in the presence of ethanol (100 mL) as a solvent and 0.01% (by weight) of p-toluene sulphonic acid as a dehydrating agent. The reaction mixture was allowed to cool overnight and then filtered. The products were recrystallized twice from ethanol, washed with water, and dried under vacuum at 60 °C to afford crystals of (1E,1′E)-1,1′-(1,4-phenylene)bis(N-(benzo[d]thiazol-2-yl)methanimine). Which was then refluxed with (0.2 mol) of different alkyl halides (R = C_6_H_13_ & C_12_H_25_ & C_18_H_37_) in acetone for 24 h. The reaction mixture was allowed to cool overnight and then filtered. The products were recrystallized twice from acetone, washed with water, and dried under vacuum at 60 °C to afford crystals of 2,2′-(1E,1′E)-1,4-phenylenebis(methaneylylidene) bis(azaneylylidene))bis(3-hexylbenzo[d]thiazol-3-ium)bromide (**TBC6**); 2,2′-(((1E,1′E)-1,4 phenylenebis(methaneylylidene))bis(azaneylylidene))bis(3-dodecylbenzo[d]thiazol-3-ium) bromide (**TBC12**) and 2,2′-(((1E,1′E)-1,4-phenylenebis(methaneylylidene))bis(azaneylylidene)) bis(3-octa decylbenzo[d]thiazol-3-ium) bromide (**TBC18**) as shown in Scheme 1.

### Characterization of TCB Gemini cationic surfactants

The structure of all synthesized surfactants (**TBC6, TBC12 and TBC 18**) was confirmed using a Thermo-Fisher Scientific Nicolet iS10 FT-IR spectrometer, United States with a range of 400–4000 cm^−1^. All solid surfactants were combined with potassium bromide pellets. Additionally, ^1^HNMR spectroscopy was used to support the conformation of all surfactant structures, where the solid surfactants are dissolved in DMSO solvent as part of sample preparation. Chemical shifts in ppm of nuclear spin of functional groups were then calculated using a 500 MHz JNM-ECA series FT-NMR, United States.

### Solvation measurements

Stock solutions of all studied surfactants (TBC6, TBC12, and TBC18) were prepared at a concentration of 0.001 M in 15% DMSO-water solvent. Salt solutions including NaCl, NaBr, NaI, CoCl_2_, MnCl_2,_ and CuCl_2_ were prepared at two concentrations (0.01 M and 0.001 M) in 15% DMSO-water solvent.

#### Conductivity measurement

4510 JENWAY CONDUCTIVITY METER 0–1.999S, United Kingdom (± 0.050 µS cm^−1^) and temperature accuracy ± 0.1 °C was calibrated using standard KCl solution and measured cell constant equal to 1 cm^−1^ [34]. The temperature was maintained and remained constant with the aid of the recirculation thermostat “ultraterm 200” adjustable temperatures from ambient + 5 °C to 200 °C (± 0.2 °C) (JP Selecta, Spain). A specific volume of each surfactant (0.5 mL or 1 mL) was added to 15% DMSO-water solvent in the absence and presence of different concentrations of inorganic salts with homogeneous mixing. Conductivity measurement was performed using an epoxy-bodied Conductivity Electrode at 298.15 K.

#### Refractive index

After the addition of a specific volume of all studied surfactants (0.5 mL or 1 mL) to 15% DMSO-water solvent in the absence and presence of different concentrations (0.001 M and 0.01 M) of inorganic salts at 298.15 K. The Mettler Toledo refractometer (± 0.0001), United States was used to determine the refractive index of the solutions by putting one drop of the solution on the center of the prism and then determining the displayed results [35].

#### Density

Density measurements were performed using one milliliter from the pure 15% DMSO-water solvent and the 15% DMSO-water solvent after adding a specific volume of each surfactant (1 mL). A Balance Digital Electronic (± 0.1 mg), United States was used to measure the weight of all previously mentioned solutions.

#### Molal volume

Molal volume measurement is a theoretical measurement of CMC of all surfactants from the data observed from density for pure solvent 15% DMSO-water solvent in the absence and the presence of different surfactant volumes [36].

#### Surface tension

Attention optical tensiometer theta lite, Biolin scientific, China (Measuring range 0.01–2000) (Accuracy ± 0.01 mN/m) with one attention software was used to measure surface tension (γ) through the contact angle of different concentrations of all examined surfactants. By detecting the diameter of the pendant drop of each surfactant solution for 30 s and then automatically measuring its surface tension [37].

## Results and discussion

### Structure confirmation of synthesized Gemini cationic surfactants

The chemical structure of the synthesized TBC Gemini cationic surfactants was confirmed using ^1^HNMR spectroscopy and IR spectroscopies.

#### ^1^HNMR chemical shift

**Fig. 1:**
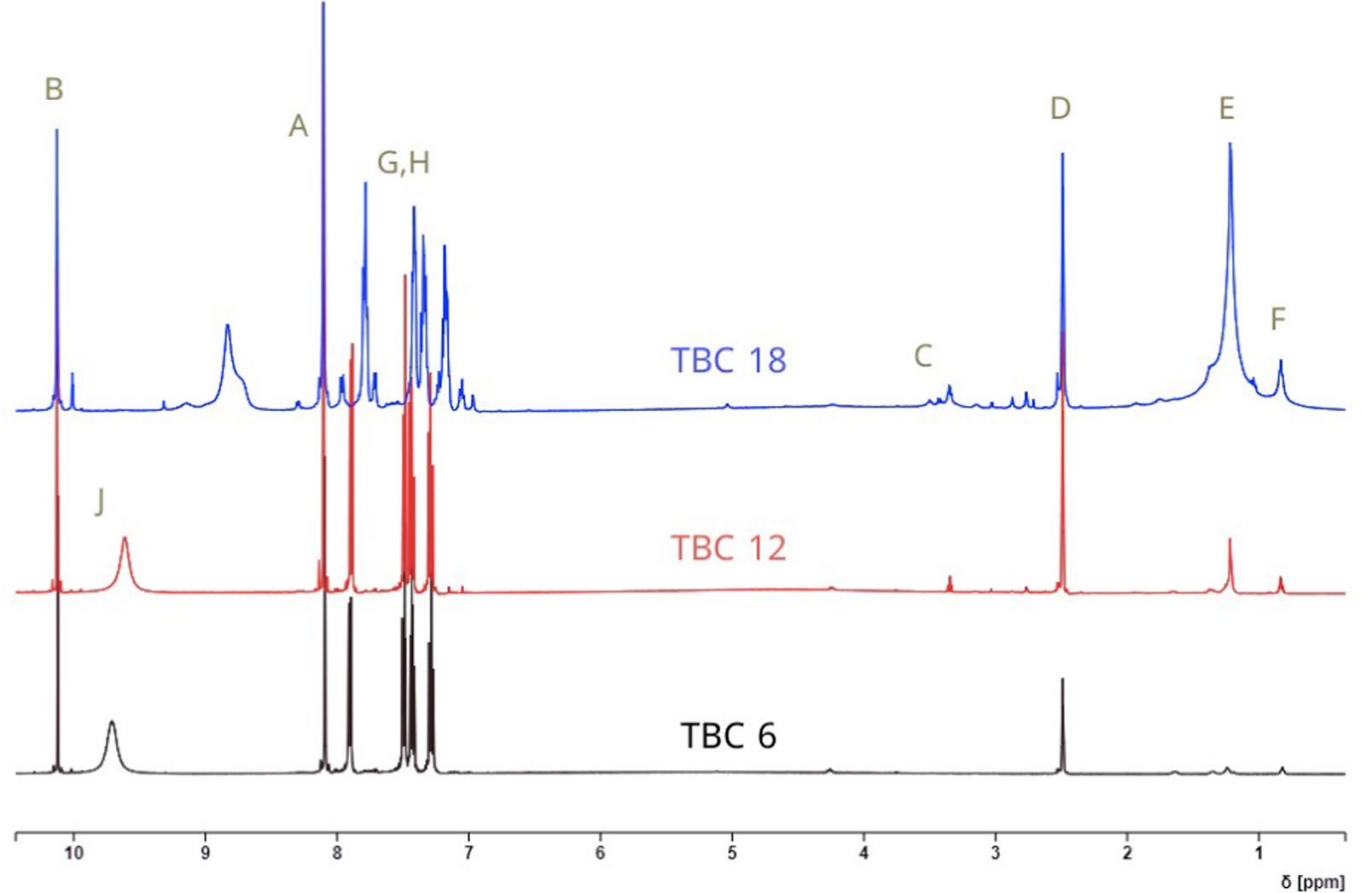
^1^HNMR spectrum of all surfactants in DMSO solvent

The chemical shifts in ppm for all surfactants were shown in Fig. 1 while the functional groups appeared in Structure 1 with symbol (a–j). The peaks at 0.84–0.85 (s, 1H, (f) –CH_3_); 1.02–1.36 (t, 6H, (e) –CH_2_); 2.49 (d, 4H, (d) –CH_2_); 3.49–3.52 (s, 2H, (c) –CH_2_); 10.12–10.15 (s, 1H, (b) –N = CH-R); 8.02–8.1 (s, 2H, (a) –CH); 7.48–7.88 (t, 3H, (g, h) –CH); 9.61–9.71 (s, H, (j) –CH).

**Structure 1:**
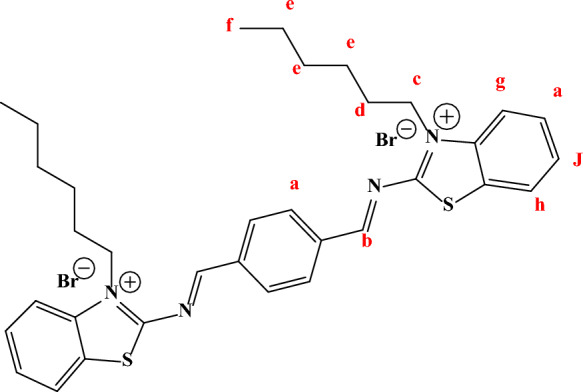
Chemical structure of TBC 6 with numbered at different positions (**a–h**)

#### IR analysis

**Fig. 2:**
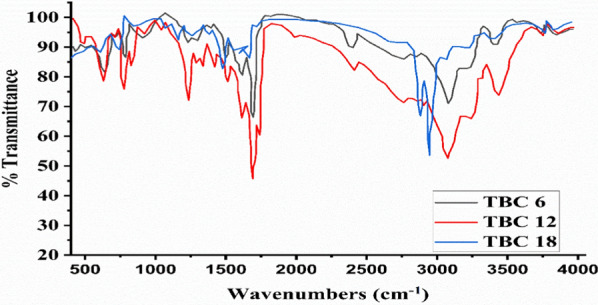
IR spectra for All Gemini cationic surfactants TBC 6, TBC 12, and TBC 18

The IR spectrum for (TBC6, TBC12, and TBC18) Gemini cationic surfactants is represented in Fig. 2. The absorption band of C–Br at 620–623 cm^−1^, aliphatic symmetric CH at 2850 cm^−1^, symmetric bending CH_2_ at 1464 cm^−1^, symmetric bending CH_3_ at 1375 cm^−1^, rock –(CH_2_)_n_– at 752 cm^−1^, aromatic amine C–N^+^ at 1202 cm^−1^, aromatic imine C=N 1691 cm^−1^, aromatic bending CH at 3042 cm^−1^, aromatic C=C at 1775 cm^−1^. While DMSO used as a solvent in sample preparation showed an absorption band at 1010 cm^−1^.

### Solvation indication

#### Critical micelle concentration measurement

**Fig. 3:**
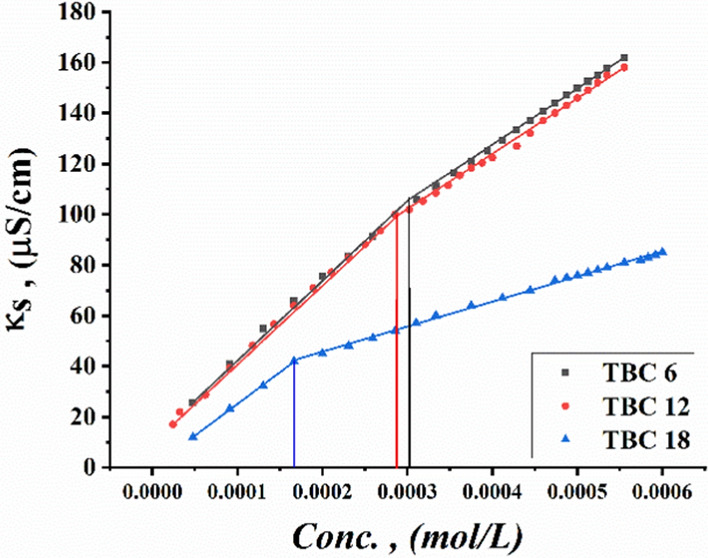
Conductivity vs. Molar Concentration for all surfactants TBC 6, TBC 12, and TBC 18 in 15% DMSO-water at 298.15K

**Fig. 4:**
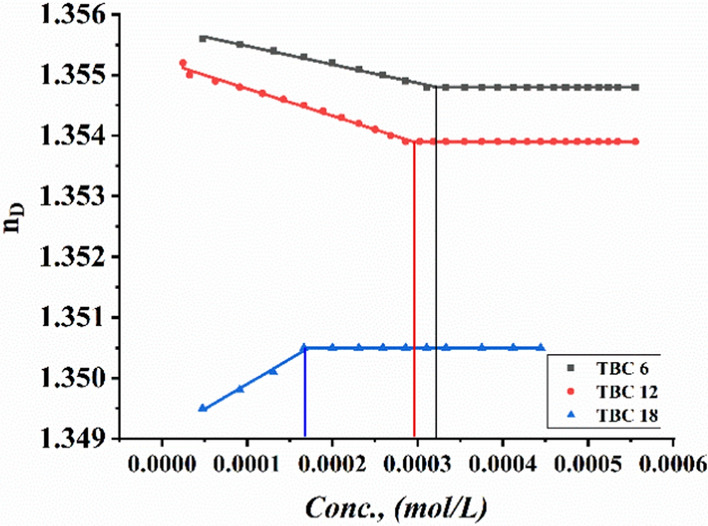
Refractive index vs. Molar Concentration for all surfactants TBC 6, TBC 12, and TBC 18 in 15% DMSO-water at 298.15K

**Fig. 5:**
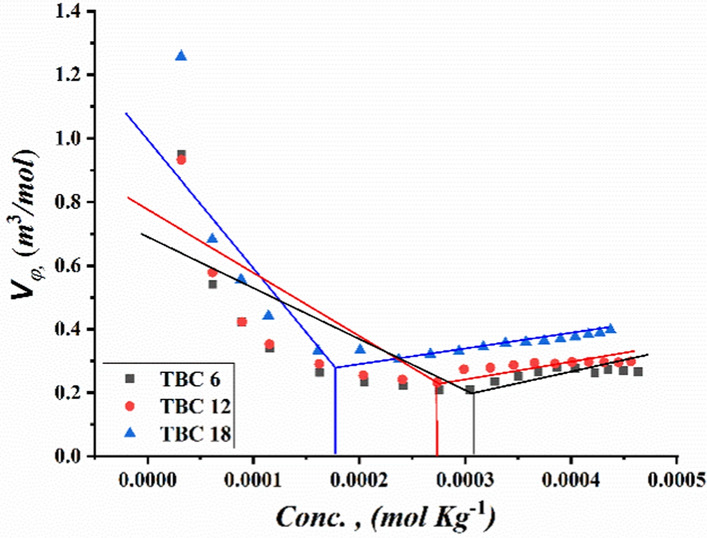
Molal Volume vs. Molal Concentration for all surfactants TBC 6, TBC 12, and TBC 18 in 15% DMSO-water at 298.15K

**Fig. 6:**
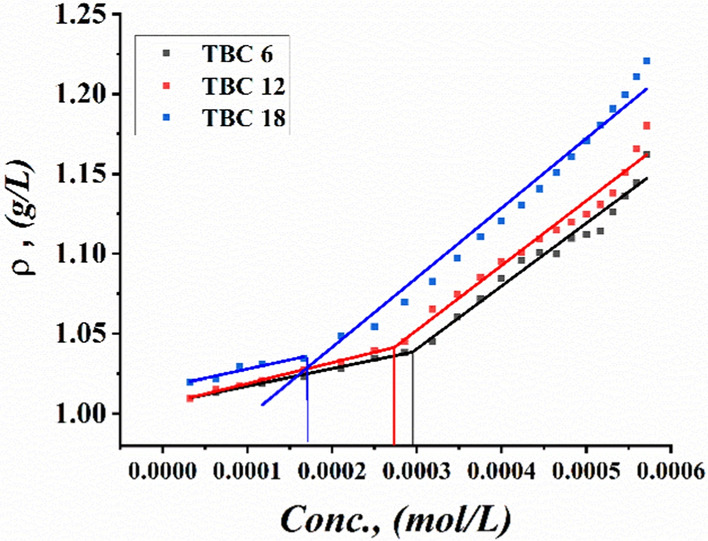
Densities vs. Molar Concentration for all surfactants TBC 6, TBC 12, and TBC 18 in 15% DMSO-water at 298.15K

**Fig. 7:**
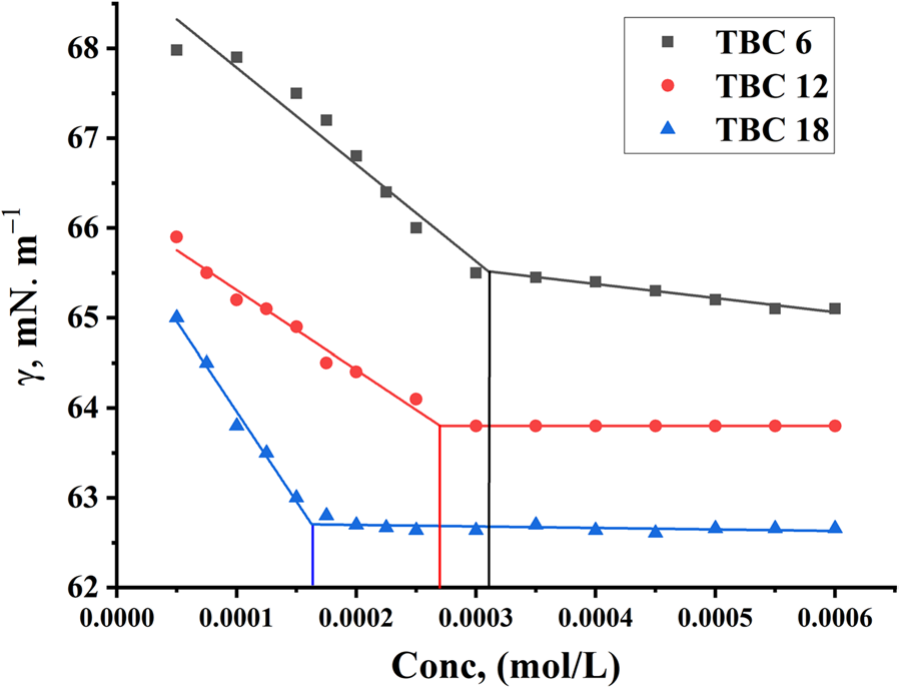
Surface tension vs. Molar Concentration for all surfactants TBC 6, TBC 12, and TBC 18 in 15% DMSO-water at 298.15K

Critical micelle concentrations of all synthesized surfactants TBC 6, TBC 12, and TBC 18 were measured by plotting various parameters, including specific conductivity, refractive index, density, molal volume, and surface tension against the concentration of the solutions after each addition of 0.001 M of each surfactant to 15% DMSO-water solvent at 298.15 K as shown in Figs. 3, 4, 5, 6, 7. The CMC values reported by each technique are summarized in Table 1.

**Table 1:** CMC values of all surfactants in 15% DMSO-water solvent

Surfactant abbreviation	CMC				
	Conductivity (mol L^−1^)	Refractive index (mol L^−1^)	Density (mol L^−1^)	Molal volume (mol kg^−1^)	Surface tension (mol L^−1^)
TBC 6	0.000302	0.000309	0.000294	0.000300	0.000312
TBC 12	0.000282	0.000286	0.000270	0.000265	0.000270
TBC 18	0.000166	0.000167	0.000171	0.000177	0.000164

Standard uncertainties (u) of CMC is = 0.00002 mol L^−1^

The Specific conductivity tends to increase with increasing mobility of the free monomers and dimers of each surfactant solvated in a 15% DMSO-water solvent [38]. There is a sharp decrease in the conductivities of all studied surfactant solutions which may be related to a decrease in the diffusion rate of each surfactant under study solvated in the same solvent. This is due to the onset of micelle formation for all surfactants TBC 6, TBC 12, and TBC 18 as shown in Fig. 3. The decrease in refractive index of TBC 6 and TBC 12 in 15% DMSO-water solvent shown in Fig. 4 is related to an increase in hydrophobic solvation between hydrocarbon chains bound to surfactant and 15% DMSO-water [39].

The molal volume of all surfactant solutions was indicated to decrease with the addition of each surfactant to 15% DMSO-water solvent before CMC. This is related to a decrease in solvent molecules bound to the hydrocarbon chains of each surfactant. After reaching CMC, it was observed that the molal volume of all studied surfactants was observed to be constant as shown in Fig. 5. This is related to the formation of micelles between the individual surfactant molecules. In contrast, the solvent molecules surrounding each surfactant are removed [40].

The densities of all studied surfactants tend to increase with each addition of all surfactants under study to 15% DMSO-water solvent as shown in Fig. 6. This is related to an increase in the molecular weight of each surfactant added to 15% DMSO-water solvent. The molecular weights of all surfactants examined were arranged in the following order: TBC 18 < TBC 12 < TBC 6 [41].

Surface tension measurement serves as a common method for determining the CMC where Gemini cationic surfactants adhere to the solution-air interface forming a monolayer. This layer is used to reduce the cohesive forces among solution molecules [42]. As illustrated in Fig. 7, the surface tension coefficient of the surfactant solutions decreases with increasing surfactant concentration till reaching their CMC [43]. However, this technique was revealed ineffectively to accurately measuring the aggregation properties of all studied Gemini cationic surfactants. This restriction might result from the solvent composition, a 15% DMSO-water mixture, that is utilized to dissolve the surfactants TBC 6, TBC 12, and TBC 18 under investigation. Polar aprotic solvent dimethyl sulfoxide (DMSO) with a high Dielectric constant may reduce the surface activity of surfactants [44]. Furthermore, disables the surface activity of surfactants by damaging their micellar structure [45].

**Fig. 8:**
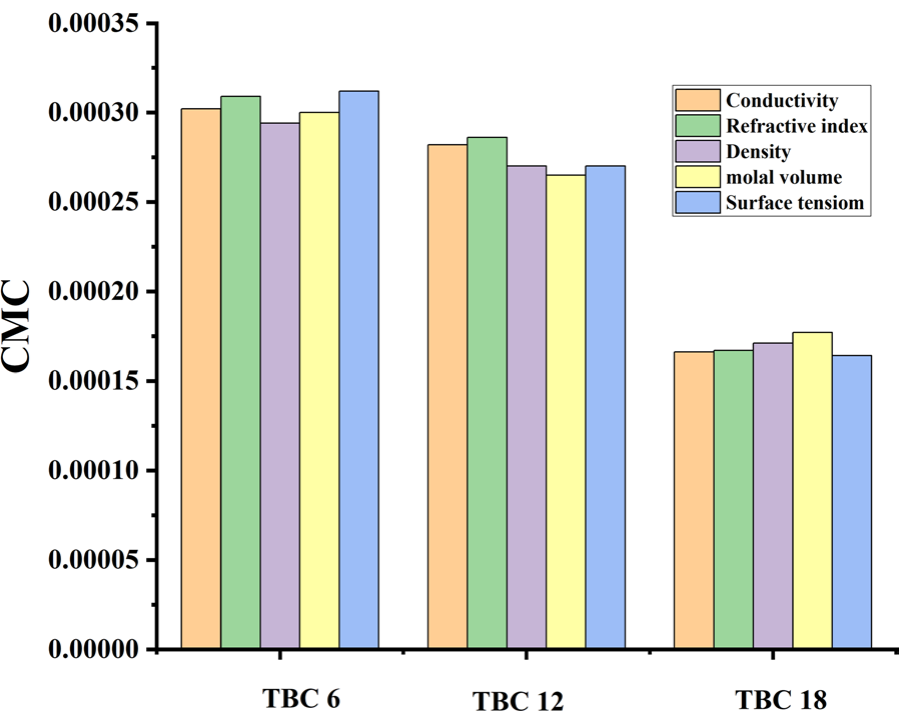
Comparison between CMC measuring from different techniques

Comparison between the critical micelle concentration of all investigated surfactants TBC 6, TBC 12, and TBC 18 in 15% DMSO-water solvent at 298.15 K was indicated as shown in Fig. 8. There was good agreement between the measuring of CMC all surfactants in the study indicated by all techniques. It was stated that the critical micelle concentration of all techniques is arranged in order TBC18 < TBC12 < TBC6.

#### Thermodynamics parameters from conductivity technique

Molar conductivity (ʌ) for all examined surfactants in 15% DMSO-water solvent at 298.15 K was calculated from Eq. (1)

1 $$ \Lambda = \frac{{1000 \times \kappa_{s} }}{C} $$

While limiting molar conductivity ($$\Lambda_{0}$$$$)$$ was estimated from Eq. (2)

2 $$ \Lambda = \Lambda_{0} - BC^{1/2} $$

binding constant for Counterions (β) was calculated by measuring the degree of ionization (α) of all surfactants in pure 15% DMSO-water solvent according to Eq. (3).

3 $$\beta =1-\propto =1-\frac{{S}_{2}}{{S}_{1}}$$

where S_2_/S_1_ represents the ratio between the slope of post-micelles to pre-micelles.

Calculation of Gibbs free energies of micellization (${\Delta G}_{mic}$) for monomer and dimer in 15% DMSO-water solvent were indicated with Eqs. (4, 5).

4 $${\Delta G}_{mic}=\left(2-\alpha \right)RT\text{ln} [CMC]$$

5 $${\Delta G}_{mic (dimer)}=\left(3-2\alpha \right)RT\text{ln}[CMC]$$

where; R is gas constant and T is absolute temperature under study = 298.15K.

Gibbs free energies for an association of all surfactants under study (${\Delta G}_{a}$) in 15% DMSO-water solvent were indicated using Eq. (6).

6 $${\Delta G}_{a}=-2.303 RT\text{log}{K}_{a}$$

where; the K_a_ association constant for all surfactants under different conditions was calculated with helping of Shedlovsky Eq. (7) [46].7where;  is a Shedlovsky function that can be calculated from Eq. 8 and Eq. 9, ${\gamma }_{i}$ is the activity coefficient, (Λ) is molar conductivity, (C) is a CMC concentration of all studied surfactants and (Λ_0_) is limiting molar conductivity.89where; (A) is the Onsager coefficient [47] equal $8.2 \times 10^{5} (\Lambda_{0} /\varepsilon T)^{3/2} + (82/\eta_{0} ) (\varepsilon T)^{1/2}$, (ε) is the dielectric constant, (T) is the absolute temperature, and $\left( {\eta_{0} } \right)$ is the viscosity of the surfactant solution.

The counterion binding constant and association constant of surfactants in 15% DMSO-water solvent tended to increase in the order TBC 6 > TBC 12 > TBC 18 as shown in Table 2. This increase follows the increase in the hydrocarbon chain length of the surfactants [48]. Gemini cationic surfactant TBC 18 was indicated to have the highest values of association and binding constant and so the largest micelle in 15% DMSO-water solvent [49, 50].

**Table 2:** Thermodynamics parameters from conductivity measurement

Sur. name	$\propto$	$\upbeta$	${\Delta G}_{mic. monomer}$(kJ mol^−1^)	${\Delta G}_{mic.dimer}$(kJ mol^−1^)	${K}_{a}$	${\Delta G}_{a}$(kJ mol^−1^)
TBC 6	0.7135	0.2865	− 25.826	− 31.60	5653.880	− 22.50
TBC 12	0.5620	0.4380	− 29.073	− 38.01	12,586.78	− 24.58
TBC 18	0.3989	0.6011	− 34.519	− 47.51	307,900.6	− 32.91

Standard uncertainties (u) of α and β are = 0.002

Gibbs free energies of association and micellization of all studied surfactants in 15% DMSO-water solvent showed an increase in the following arrangement: TBC 18 > TBC 12 > TBC 6. This may be related to an increase in micelle formation with an increase in the hydrocarbon chain length of surfactants [51]. The negative value of the Gibbs free energies for micellization and association was found to be spontaneous for these processes [52]. Different models were used to measure Gibbs free energies for studied Gemini cationic surfactants including the phase separation model [53] and pseudo-phase separation model [54]. Both models proved that the increase in hydrocarbon chain length led to an increase in micellization as a following arrangement TBC 18 < TBC 12 < TBC 6.

The decrease in the degree of ionization (α) has been attributed to a shift in charge density at the micelle surface as the chain length increases. A longer chain length results in a higher degree of aggregate compactness, and the “head groups” tend to approach more, indicating that more counter ions are drawn to the Stern layer surrounding the “heads” and lowering the micelle ionization degree [55].

#### Molal volume

From the measurement of the density of the pure solvent (${\rho }^{o}$) and the density of the solution after each addition of all surfactants to 15% DMSO-water solvent ($\rho$), the molal volume $V_{\varphi }$ of all investigated surfactants was calculated as in Eq. (10).

10 $$ V_{\varphi } = \frac{M}{\rho } - \frac{1000}{m} \left[ {\frac{1}{{\rho^{o} }} - \frac{1}{\rho }} \right] $$

where; M is the molecular weight of each surfactant and m is the molal concentration of each surfactant in the solvent.

The packing density ($P$) had a constant value of 0.661 for the large molecules. The van der Waals volume (${V}_{w}$) and electrostriction volume (${V}_{E}$) were calculated from Eq. (11).

11 $$ V_{w} = P. V_{\varphi } = V_{E} + V_{\varphi } $$

**Fig. 9:**
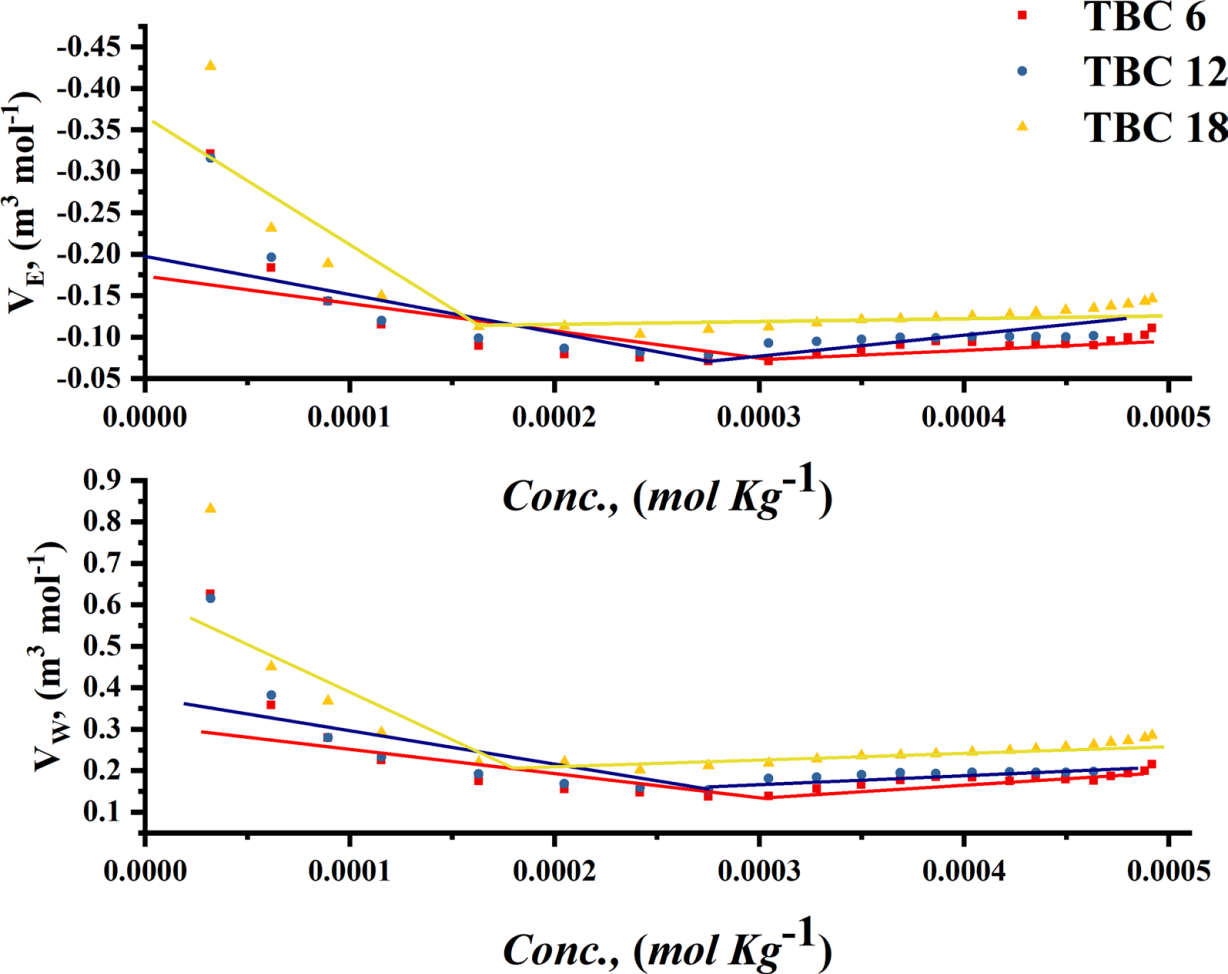
The relationship between molal concentration against Van der Waals Volume (V_w_) and electrostriction Volume (V_E_) for all surfactants

The changes in electrostriction volume ${V}_{E}$ and van der Waals volume ${V}_{w}$ for all investigated surfactants are shown in Fig. 9.

The interaction between all studied surfactants (**TBC6, TBC12, and TBC18**) and 15% DMSO-water solvent was shown to decrease with increasing surfactant concentrations [56]. This may be related to a decrease in solvation between the hydrophobic hydrocarbon chains of surfactants and the solvent molecules surrounding these chains [57, 58]. The decrease in solvation leads to a reduce the surface area of interaction and enhanced steric hindrance between surfactant molecules leading to decreasing in the Van der Waals volume [59, 60]. The chemical structure of surfactants, including the nature of their hydrophilic and hydrophobic groups and the high polarity of 15% DMSO-water solvent influences how Gemini cationic surfactants respond to electric fields [61]. The presence of an electric field can alter the size, shape, and stability of these aggregates. As shown in Fig. 9, the electrostriction volume of all studied surfactant solutions decreases which may be related to that the micelles become more stable and reduce their ability to undergo significant volume changes in response to electric fields [62].

#### Modeling of densities

From the calculation, the density and molal volumes of all surfactants in 15% DMSO-water at different concentrations at 298.15K. This modeling is indicated according to the Setschenow relationship shown in Eq. (12).

12 $$\text{log}\frac{{\rho }^{o}}{\rho }=KC$$

where; ${\rho }^{o}/\rho$ is the ratio between the density of 15% DMSO-water solvent and each addition of all surfactants (TBC6, TBC12, and TBC18) into the solvent.

**Fig. 10:**
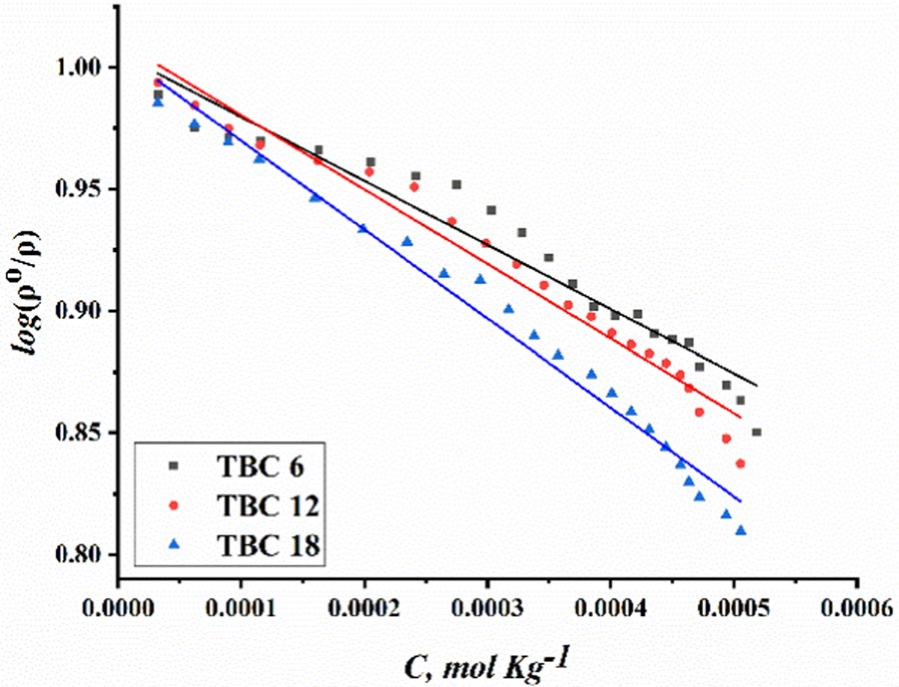
Modeling changes in densities of all surfactants TBC 6, TBC 12, and TBC 18 in 15% DMSO-water solvent

The densities of the surfactant solutions (TBC6, TBC12, and TBC18) were reported to increase with the addition of each surfactant in 15% DMSO-water solvent at 298.15 K as shown in Fig. 10. This could be related to the increase in molecular weight of surfactant in the following arrangement: TBC18 < TBC12 < TBC6. It is stated that the Setschenow constant increases with increasing hydrocarbon chain length as summarized in Table 3.

**Table 3:** The Setschenow parameter

Surfactant abbreviation	K	R^2^
TBC 6	− 263.09	0.96
TBC 12	− 305.64	0.98
TBC 18	− 364.84	0.96

The negative values of the Setschenow constant indicate an increase in the density of the solutions after adding the investigated surfactants TBC 6, TBC 12, and TBC 18 to 15% DMSO-water solvent. An increase in the values of the setschenow constant indicates the direction of density arrangement in the following TBC18 < TBC12 < TBC6. While the Gemini cationic surfactant TBC18 has the greatest value as it has the largest molecular weight [63].

#### Refractive index

Molar refraction (${R}_{m}$) and atomic polarization (${P}_{A}$) for all newly Gemini cationic surfactants TBC 6, TBC 12, and TBC 18 were calculated from data observed from refractive indices measurement at a fixed concentration in 15% DMSO-water solvent at 298.15K by using Eq. (13).

13 $${R}_{m}=\frac{{V}_{\varphi }({n}^{2}-1)}{\frac{{P}_{A}}{1.05}+2}$$

The polarizability (${\alpha }_{D}$) of all surfactants TBC 6, TBC 12, and TBC 18 in 15% DMSO-water solvent at 298.15K was calculated from Eq. (14) by using the Avogadro number (N).

14 $${\alpha }_{D}= \frac{{3V}_{\varphi }({n}^{2}-1)}{{4N\pi (n}^{2}+2)}$$

when comparing the molar refraction and polarizability values of all surfactants (TBC6, TBC12, and TBC18) as shown in Table 4, it was observed that the data increased as the hydrocarbon chain of the surfactants increased. This is related to the increasing strength of the hydrophobic interaction between the hydrocarbon chain of surfactants and the surrounding 15% DMSO-water solvent molecules. The surfactant TBC 18 was reported to have the highest values in molar refraction and polarizability, thus ensuring the strongest hydrophobic solvation [64].

**Table 4:** refractive index (n_D_), molar refraction (${R}_{m}$), atomic polarizability (P_A_), and the polarizability (${\alpha }_{D}$) of TBC 6, TBC 12, and TBC 18 at the same concentration in 15% DMSO-water at 298.15K

Molal Conc.	Surfactants	n_D_	${R}_{m}$(m^3^ mol^−1^)	${P}_{A}$(m^3^ mol^−1^)	${\alpha }_{D}$(m^3^) E-26
0.00013	TBC 6	1.3553	0.0742	1.9287	2.94
	TBC 12	1.3548	0.0769	1.9273	3.05
	TBC 18	1.3505	0.0951	1.9150	3.77

Standard uncertainties (u) of n_D_ = 0.02_,_ R_m=_0.001, P_A_ = 0.03, α_D_ = 0.04

##### Enhancing aggregation properties under salts effect

##### Detection of CMC

##### Conductometric techniques

**Fig. 11:**
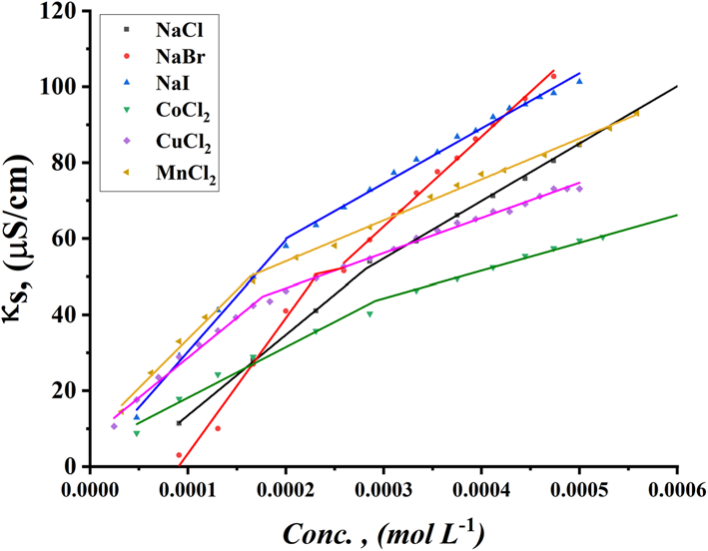
Conductivity vs. concentration of 0.001 M solution of different salts solution after addition of TBC 6 surfactant at 298.15 K

**Fig. 12:**
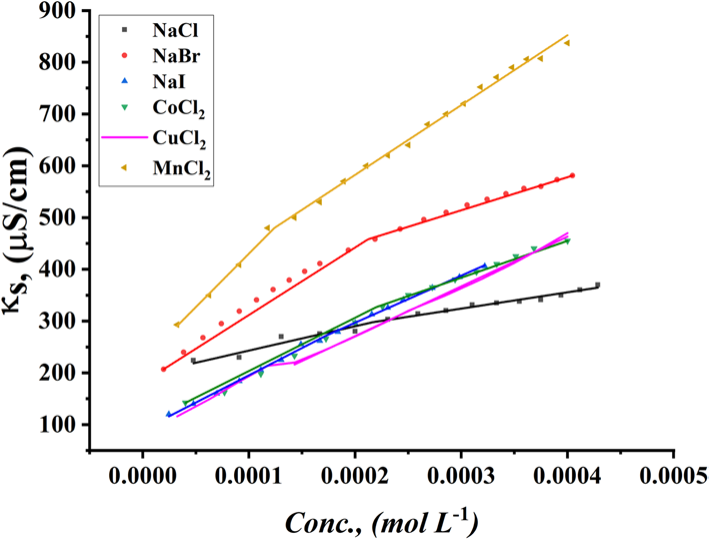
Conductivity vs. concentration of 0.01 M solution of different salts solution after addition of TBC 6 surfactant at 298.15 K

The effect of the addition of various inorganic salts on the CMC of all studied surfactants was indicated by conductometric measurement as shown in Figs. 11, 12 for TBC 6 surfactant and Figs. S1-S4 for both TBC 12 and TBC 18 respectively. The relationship between solution conductivity and concentration of all studied surfactants in 15% DMSO-water solvent in the presence of two different concentrations (0.001 and 0.01 M) of six different inorganic salts, including NaCl, NaBr, NaI, CoCl_2_, CuCl_2,_ and MnCl_2_. The effect of different salts on the critical micelle concentration of all examined surfactants is summarized in Table 5.

**Table 5:** CMC of all surfactants TBC 6, TBC 12, and TBC 18 with different salt concentrations (0.001M and 0.01M) in 15% DMSO-water solvent at 298.15K with conductometric technique

Salt Conc. mol L^−1^	CMC (mol L^−1^)							
	Solvent	15% DMSO-water without the addition of salts	15% DMSO-water with the addition of different salts					
	Additives		NaCl	NaBr	NaI	CoCl_2_	CuCl_2_	MnCl_2_
0.001	TBC 6	0.000302	0.000285	0.000244	0.000195	0.000291	0.000131	0.000168
	TBC 12	0.000282	0.000179	0.000162	0.000181	0.000248	0.000146	0.000159
	TBC 18	0.000166	0.000172	0.000124	0.000143	0.000210	0.000113	0.000124
0.01	TBC 6	0.000302	0.000224	0.000211	0.000177	0.000219	0.000176	0.000121
	TBC 12	0.000282	0.000155	0.000147	0.000141	0.000177	0.000127	0.000094
	TBC 18	0.000166	0.000139	0.000124	0.000104	0.000122	0.000058	0.000058

Standard uncertainties (u) of CMC = 0.00002 mol l^−1^

The CMC values of all surfactants tended to decrease with the addition of salts compared to their values in 15% DMSO-water solvent as shown in Table 5. The addition of salts increased the micelle formation rate for all surfactants studied due to the salting out effect, where added salts interacted with 15% DMSO-water solvent leaving surfactants with lesser solvent molecules around them [65]. Due to the salting-out effect, the hydrocarbon chains of all investigated surfactants TBC 6, TBC 12, and TBC 18 can interact more easily to form micelles at lower concentrations [66]. According to this concept, a larger cation size has a larger solvated shell which reduces the effective size and charge density of the counter ions available for interaction with the surfactant head groups [67]. The radius of cations in (A°) Mn^2+^ = 0.83, Co^2+^ = 0.75, and Cu^2+^ = 0.73. It is stated that the effect of reducing CMC follows the following trend CuCl_2_ > MnCl_2_ > CoCl_2_.

The radius of salts influences the internuclear separation, ionic strength, ionization potential, lattice energy, and solubility of salts in various media [68]. Salts with higher lattice energy have more ability to attract solvent molecules and so increase micellization. The lattice energy for cations was indicated to be 2532, 2688, and 2804 kJ/mol for MnCl_2_, CoCl_2,_ and CuCl_2_. It is stated that copper chloride has the greatest ability to reduce the CMC of all studied surfactants.

Furthermore, the presence of salts in the solvent increases the ionic strength of surfactant solutions [69]. This increase led to a reduction in the repulsion forces in surfactant head groups [70]. By comparing the aggregation behavior of all surfactants under the influence of all salts used, the decrease in the CMC of all surfactants is associated with an increase in the radius of the counter ions. The radii of the anions used in this study had values of Clˉ = 1.81 A°, Brˉ = 1.96 A°, and Iˉ = 2.2 A° while for [71–73]. As the size of the anions or cations increased, the ionic strength of the solution increased. This can lead to a reduction in the repulsion force between the head groups of all surfactants. This effect in turn causes the micellization of all surfactants to increase at lower concentrations. CMC for all investigated surfactants is given in ascending order as follows NaI > NaBr > NaCl.

##### Refractive index measurement

**Fig. 13:**
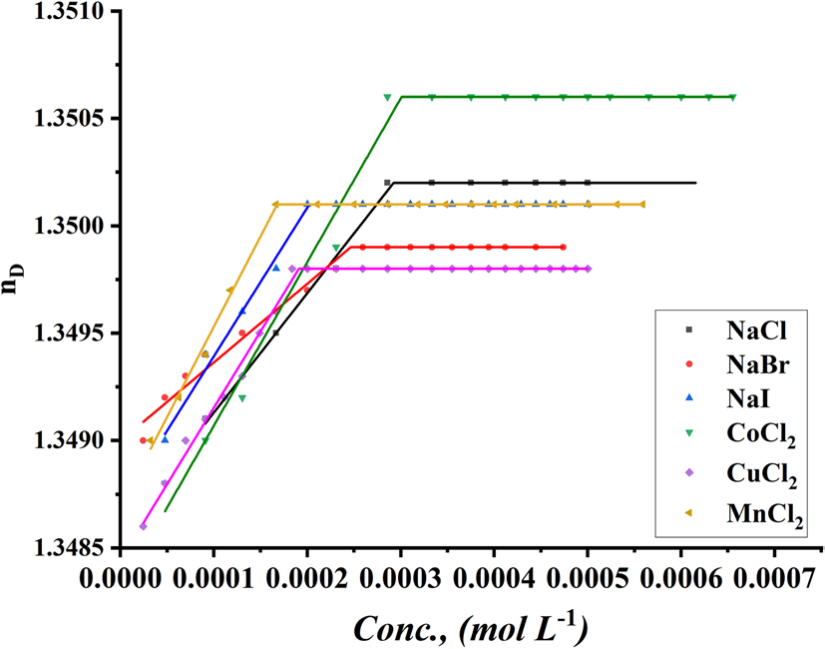
Refractive index vs. concentration of 0.001 M solution of different salts solution after addition of TBC 6 surfactant at 298.15 K

**Fig. 14:**
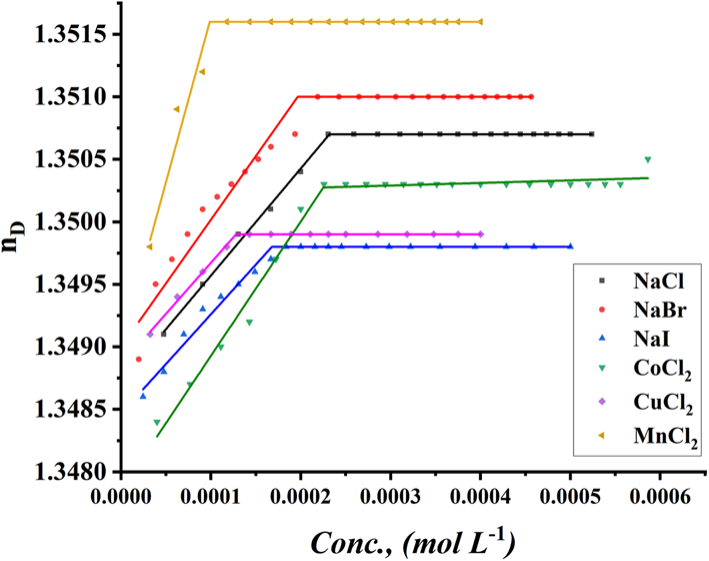
Refractive index vs. concentration of 0.01 M solution of different salts solution after addition of TBC 6 surfactant at 298.15 K

The CMC of all studied surfactants was given by adding a specific volume of all surfactants to a specific volume of two different concentrations of six inorganic salts. The CMC of all indications was performed using a refractometer to determine the refractive index for each addition of surfactant to different media, including all six inorganic salts, with the refractive index of all surfactants being 0.001 and 0.01 M NaI, NaBr, NaCl, MnCl_2_, CuCl_2,_ and CoCl_2_ plotted against the concentration of the surfactant solution, as shown in Figs. 13, 14 for TBC 6 surfactant and Figs. S5-S8 for TBC 12 and TBC 18 respectively.

Various inorganic salts were shown to have the same effect on lowering the CMC of all surfactants studied as previously demonstrated in conductometric measurements, proving the salting out of the studied surfactant from 15% DMSO-water solvent at 298.15 K as shown in Table 6. It is stated that various inorganic salts condense in the stern layer around the head groups of each surfactant [74].

**Table 6:** CMC of all surfactants in different salt solutions with 0.001M and 0.01M solution with 15% DMSO-water solvent at 298.15K with refractive index techniques

Salt Conc. mol L^−1^	CMC (mol L^−1^)							
	Solvent	15% DMSO-water without the addition of salts	15% DMSO-water with the addition of different salts					
	Additives		NaCl	NaBr	NaI	CoCl_2_	CuCl_2_	MnCl_2_
0.001	TBC 6	0.000309	0.000290	0.000248	0.000168	0.000300	0.000126	0.000169
	TBC 12	0.000286	0.000174	0.000167	0.000142	0.000240	0.000150	0.000141
	TBC 18	0.000167	0.000188	0.000149	0.000152	0.000207	0.000115	0.000113
0.01	TBC 6	0.000309	0.000230	0.000198	0.000168	0.000227	0.000187	0.000098
	TBC 12	0.000286	0.000140	0.000149	0.000144	0.000169	0.000132	0.000091
	TBC 18	0.000167	0.000149	0.000111	0.000094	0.000119	0.000059	0.000050

Standard uncertainties (u) of CMC = 0.00002

The size of the micelle formed depends on the size of the counter ions adsorbed on the surface of the head groups of each surfactant. Iodide counter anions were shown to have the greatest effect when each surfactant was salted out in 15% DMSO-water solvent at 298.15 K. The iodide anion was reported to have the largest radius with the largest CMC reduction [28].

The CMC of all surfactants decreased with increasing salt concentration used in this study. It was indicated that the surfactants in 15% DMSO-water solvent with a salt concentration of 0.01M were indicated to have lower CMC than the same surfactants in 15% DMSO-water solvent with a salt concentration of 0.001M using techniques that were measured. This effect proved the relationship between the increase of multiple counter ions condensing around the head groups of all surfactants and the size of the formed micelle [23].

#### Thermodynamic parameters from conductivity measurements under the effect of salts.

Several thermodynamic parameters, including the degree of ionization, the counter ion binding constant, the association constant, and the Gibbs free energy of micellization and association were calculated as previously mentioned in Eqs. (1–9).

All degrees of ionization of each surfactant (TBC 6, TBC 12, and TBC 18) were reported to increase with the addition of all studied salts at different concentrations (0.01 M and 0.001 M) as listed in Table 7. The increase in the degree of ionization of the surfactant solutions is related to the increase in the ionic strength of the surfactant solution in the presence of all six inorganic salts. It was found that the degree of ionization of each surfactant in the presence of 0.01 M of all salts was higher than that of the same surfactant in the presence of 0.001 M of all salts [75].

**Table 7:** The degree of ionization, (α) the counter ion binding, (β) and the standard free energy of micellization, the limiting molar conductivity (Λ_0_), association constant (K_a_), and the standard free energy change of association (ΔG_a_) and micellization (ΔG_mic._) for the surfactants understudy in 0.001M and 0.01M solutions of different inorganic salts 15% DMSO –water solvent at 298.15K

Sur. name	Salt Conc. mol L^−1^	Salt name	$\propto$	$\upbeta$	${\Delta G}_{mic}$ (kJ/mol)	${\Lambda }_{0}$ S.m^2^.mol^−1^	${K}_{a}$	${\Delta G}_{a}$ (kJ/mol)
TBC 6	0.001	NaCl	0.7136	0.2864	− 26.030	2326	1,093,447	− 34.47
		NaBr	0.7467	0.2533	− 25.843	2133	1,498,273	− 35.25
		NaI	0.7934	0.2066	− 25.549	2791	1,653,112	− 35.50
		CoCl_2_	0.7829	0.2171	− 24.565	5150	2,553,115	− 36.58
		CuCl_2_	0.7666	0.2334	− 26.431	3524	1,012,979	− 34.28
		MnCl_2_	0.7807	0.2193	− 26.269	3619	1,982,866	− 35.95
	0.01	NaCl	0.9556	0.0444	− 27.756	21,579	2,518,357	− 36.54
		NaBr	0.7950	0.2050	− 26.280	23,756	1,846,096	− 35.77
		NaI	0.9548	0.0452	− 26.384	23,378	4,386,258	− 37.91
		CoCl_2_	0.8591	0.1409	− 26.832	32,209	5,341,669	− 38.40
		CuCl_2_	0.8258	0.1742	− 26.021	39,994	6,861,714	− 39.02
		MnCl_2_	0.9197	0.0803	− 25.154	41,314	2,600,565	− 36.62
TBC 12	0.001	NaCl	0.6702	0.3298	− 28.441	2326	1,266,818	− 34.84
		NaBr	0.6674	0.3326	− 28.830	2433	1,666,505	− 35.52
		NaI	0.7642	0.2358	− 26.398	2791	1,599,675	− 35.42
		CoCl_2_	0.6662	0.3338	− 27.449	4950	2,958,284	− 36.94
		CuCl_2_	0.7118	0.2882	− 28.201	3724	1,072,670	− 34.43
		MnCl_2_	0.8348	0.1652	− 25.264	2919	2,573,418	− 36.6
	0.01	NaCl	0.7396	0.2604	− 29.406	16,000	4,832,914	− 38.15
		NaBr	0.7384	0.2616	− 29.598	19,000	2,556,325	− 36.55
		NaI	0.9111	0.0889	− 29.933	14,000	5,799,868	− 38.61
		CoCl_2_	0.8077	0.1923	− 29.534	32,000	9,683,245	− 39.88
		CuCl_2_	0.5618	0.4382	− 31.984	31,000	4,700,581	− 38.08
		MnCl_2_	0.8884	0.1116	− 29.550	39,000	3,767,181	− 37.54
TBC 18	0.001	NaCl	0.5694	0.4306	− 34.737	1326	1,854,616	− 35.78
		NaBr	0.6055	0.3945	− 34.094	1433	1,954,509	− 35.91
		NaI	0.7421	0.2579	− 33.604	1791	2,474,822	− 36.50
		CoCl_2_	0.7336	0.2664	− 33.585	3050	3,010,138	− 36.98
		CuCl_2_	0.7580	0.2420	− 33.979	3524	1,673,414	− 35.53
		MnCl_2_	0.8995	0.1005	− 33.538	2919	2,895,114	− 36.89
	0.01	NaCl	0.7593	0.2407	− 34.314	12,000	5,628,836	− 38.53
		NaBr	0.6344	0.3656	− 34.449	12,000	4,114,195	− 37.75
		NaI	0.8050	0.1950	− 35.166	18,000	9,697,917	− 39.80
		CoCl_2_	0.7995	0.2005	− 36.817	34,000	14,099,602	− 40.81
		CuCl_2_	0.8108	0.1892	− 38.756	22,000	22,244,399	− 41.94
		MnCl_2_	0.9026	0.0974	− 36.536	25,000	9,185,123	− 39.75

Standard uncertainties (u) of α and β are = 0.002

It was shown that the Gibbs free energy of association and micellization increase with increasing the addition of all examined salts at the same concentration. This indicated that the formation of the micelle generated much more energy indicating the stability of the formed micelle [29]. The negative magnitude of the Gibbs free energy of both micellization and association indicates spontaneous processes [76]. The increase in the negativity of Gibbs free energy proved the increase in the formation of more stable micelles [77].

**Fig. 15:**
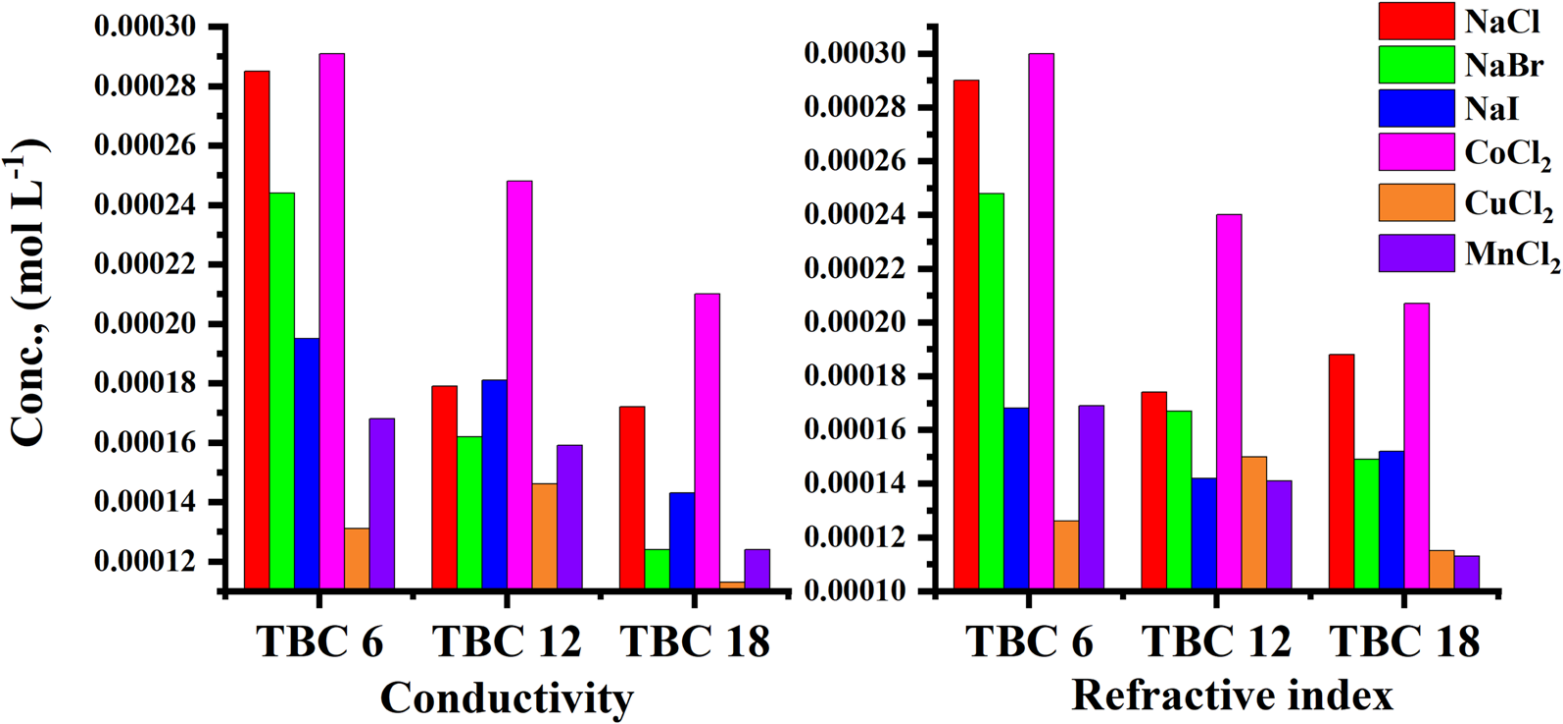
Comparison between CMC of different surfactants TBC 6, TBC 12, and TBC 18 under the effect of different salts at 0.001 M using conductivity and refractive index measurement

**Fig. 16:**
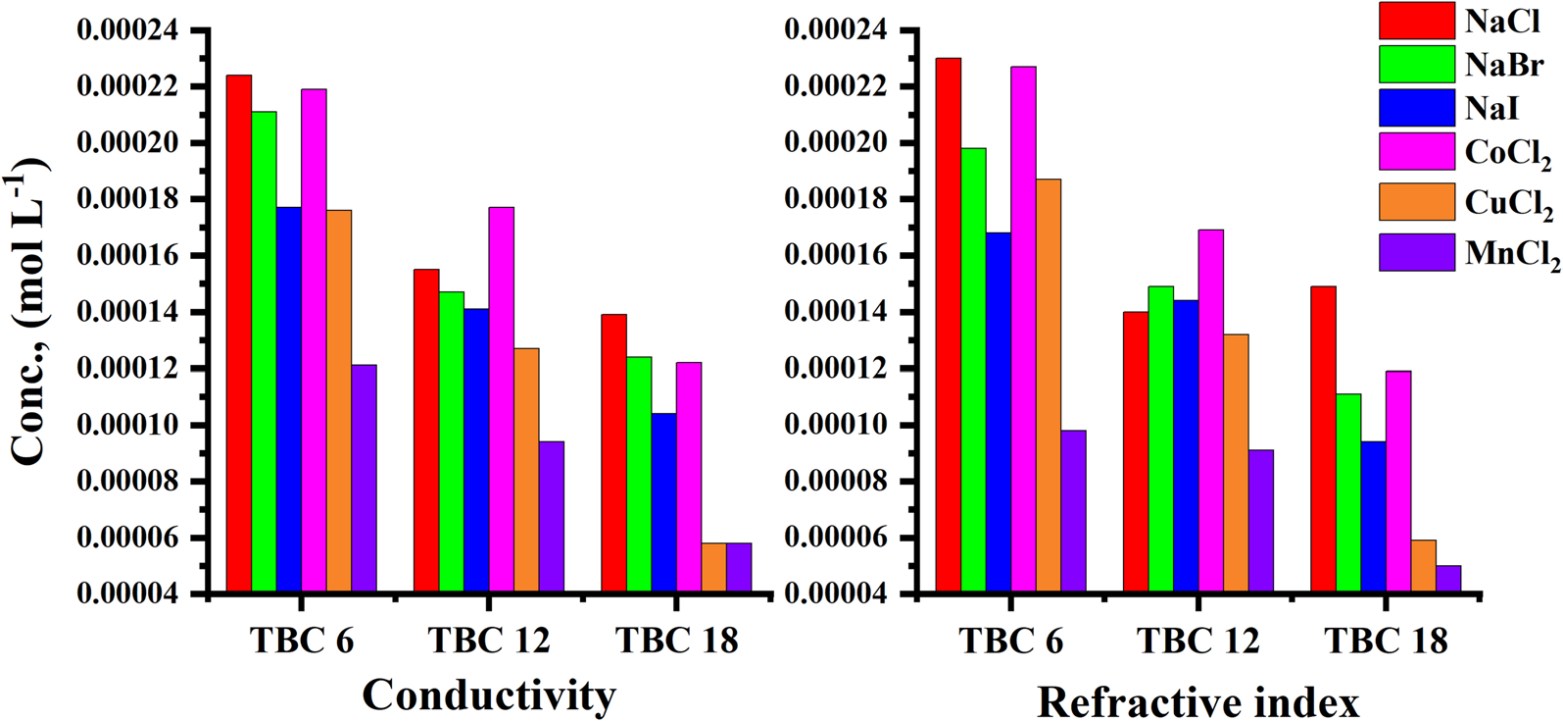
Comparison between CMC of different surfactants TBC 6, TBC 12, and TBC 18 under the effect of different salts at 0.01 M using conductivity and refractive index measurement

The effect of different inorganic salts on all surfactants under study TBC 6, TBC 12, and TBC 18 from different techniques including conductivity and refractive index measurement were compared to indicate the acceptability agreement in proving results as shown in Fig. 15 at salts concentration equal 0.001M and Fig. 16 at salts concentration equal 0.01M.

## Conclusion

The CMC values of a new series of Gemini cationic surfactants with different hydrocarbon chain lengths were detected with different techniques including conductivity, refractive index, surface tension, density, and molal volume in 15% DMSO-water solvent at 298.15 K. Raw data from different techniques indicated to have good compatibility to explain the micellization process. Counter ions binding constant (β) and association constant (K_a_) demonstrated the formation of a more stable micelle with increased surfactant concentration and hydrocarbon chain length. An increase in the negativity of micellization (ΔG_mic_) and association (ΔG_ass_) Gibbs free energies verified the increasing spontaneity of surfactant aggregation TBC 18 > TBC 12 < TBC 6. Micellization of Gemini cationic surfactants was verified by a decrease in the molal volume (${V}_{\phi }$), van der Waals volume (${V}_{w}$), and electrostriction volume (${V}_{E}$), indicating a decrease in the interaction between surfactants and surrounding solvent till reaching CMC and formation of more stable micelle. The CMC of all studied surfactants in the presence of different concentrations of salts was determined by conductivity and refractive index measurement. A decrease in CMC was indicated due to adsorption on the stern layer and a decrease in repulsion force between head groups of surfactants which indicated the following CuCl_2_ > MnCl_2_ > CoCl_2_ > NaI > NaBr > NaCl. An increase in the concentration of salts verified increasing in micellization of each surfactant as the following 0.01mol L^−1^ < 0.001 mol L^−1^. Degrees of ionization (α) for all studied surfactants indicated an increase in the presence of different inorganic salts, indicating the formation of micelle at lower concentrations. An increase in the negativity of Gibbs free energies of micellization (ΔG_mic_) and association (ΔG_ass_) in the presence of salts compared to the absence of salts indicated an increase in the stability of the micelle formed. This study contributes to understanding the design of surfactants by correlating hydrocarbon chain length, concentration, and addition of salts with surfactant properties and tailoring it to specific industrial applications such as detergency, emulsification, and corrosion inhibitor applications.

## Supplementary Information

Additional file 1.

## Data Availability

Raw data were generated at Faculty of Science, Port-Said University, Egypt. Derived data supporting the findings of this study are available from the corresponding author, Prof. Dr. Farid I. El-Dossoki, on request.
