# Supplementary material for: Synergistic enhancing of micellization and thermodynamic properties of some Gemini cationic surfactants related to benzo[d]thiazol-3-ium bromide
Source: BMC Chem. 2024 Dec 11;18(1):240. doi: 10.1186/s13065-024-01334-9 (PMC11636038; doi:10.1186/s13065-024-01334-9)
Supplement: Supplementary file 1 — Additional file 1. [file 13065_2024_1334_MOESM1_ESM.docx]

**Fig. 13 .s** Conductivity vs. concentration of 0.001 M solution of different salts solution after addition of TBC 12 surfactant at 298.15 K




**Fig. 14 .s** Conductivity vs. concentration of 0.01 M solution of different salts solution after addition of TBC 12 surfactant at 298.15 K





**Fig. 15 .s** Conductivity vs. concentration of 0.001 M solution of different salts solution after addition of TBC 18 surfactant at 298.15 K





**Fig. 16 .s** Conductivity vs. concentration of 0.001 M solution of different salts solution after addition of TBC 18 surfactant at 298.15 K





**Fig. 19 .s** refractive index vs. concentration of 0.001 M solution of different salts solution after addition of TBC 12 surfactant at 298.15 K.





**Fig. 20 .s** refractive index vs. concentration of 0.01 M solution of different salts solution after addition of TBC 12 surfactant at 298.15 K.





**Fig. 21 .s** refractive index vs. concentration of 0.001 M solution of different salts solution after addition of TBC 18 surfactant at 298.15 K.





**Fig. 22 .s** refractive index vs. concentration of 0.01 M solution of different salts solution after addition of TBC 18 surfactant at 298.15 K
